# Estimation of potential social support requirement for tuberculosis patients in India

**DOI:** 10.1093/heapol/czae065

**Published:** 2024-07-10

**Authors:** Susmita Chatterjee, Guy Stallworthy, Palash Das, Anna Vassall

**Affiliations:** Research, George Institute for Global Health, 308, Elegance Tower, Plot No 8, Jasola District Centre, New Delhi 110025, India; Faculty of Medicine, University of New South Wales, High St, Kensington, NSW 2052, Australia; Global Health Division, Bill & Melinda Gates Foundation, 500 Fifth Avenue North, Seattle, WA 98109, United States; Research, George Institute for Global Health, 308, Elegance Tower, Plot No 8, Jasola District Centre, New Delhi 110025, India; Global Health and Development, London School of Hygiene and Tropical Medicine, Keppel St, WC1E 7HT, United Kingdom

**Keywords:** Catastrophic cost, direct benefit transfer, nikshay poshan yojaya, social protection, tuberculosis, India

## Abstract

Providing social support to tuberculosis (TB) patients is a recommended strategy as households having TB patients find themselves in a spiral of poverty because of high cost, huge income loss and several other economic consequences associated with TB treatment. However, there are few examples of social support globally. The Indian government introduced the ‘Nikshay Poshan Yojana’ scheme in 2018 to provide nutritional support for all registered TB patients. A financial incentive of 500 Indian Rupee (6 United States Dollars) per month was proposed to be transferred directly to the registered beneficiaries’ validated bank accounts. We examined the reach, timing, amount of benefit receipt and the extent to which the benefit alleviated catastrophic costs (used as a proxy to measure the impact on permanent economic welfare as catastrophic cost is the level of cost that is likely to result in a permanent negative economic impact on households) by interviewing 1482 adult drug-susceptible TB patients from 16 districts of four states during 2019 to 2023, using the methods recommended by the World Health Organization for estimating household costs of TB nationally. We also estimated the potential amount of social support required to achieve a zero catastrophic cost target. At the end of treatment, 31–54% of study participants received the benefit. In all, 34–60% of TB patients experienced catastrophic costs using different estimation methods and the benefit helped 2% of study participants to remain below the catastrophic cost threshold. A uniform benefit amount of Indian Rupee 10 000 (127 United States Dollars) for 6 months of treatment could reduce the incidence of catastrophic costs by 43%. To improve the economic welfare of TB patients, levels of benefit need to be substantially increased, which will have considerable budgetary impact on the TB programme. Hence, a targeted rather than universal approach may be considered. To maximize impact, at least half of the revised amount should be given immediately after treatment registration.

Key messages

- Providing social support to tuberculosis (TB) patients is a recommended strategy as households having TB patients find themselves in a spiral of poverty because of high cost, huge income loss and several other economic consequences associated with TB treatment. However, there are few examples of social support globally. The government of India introduced a benefit scheme namely ‘Nikshay Poshan Yojana’ (NPY) on 1 April 2018, with the objective of providing nutritional support to all notified TB patients. The benefit is transferred directly to the beneficiary’s bank account to the value of 500 Indian Rupee per month for the whole duration of TB treatment (6 months for drug-susceptible TB).
- We examined the reach, amount of NPY benefit receipt at different treatment phases and by wealth quintile and the extent to which NPY alleviated catastrophic costs, and estimated the potential amount of social support required to achieve a zero catastrophic cost target by following-up a cohort of 1482 adult drug-susceptible TB patients from three groups: general population, urban slum dwellers and tea garden residents.
- We found that the uptake of the benefit was modest, with 31–54% of study participants receiving the benefit and 5–11% of study participants receiving the full amount of benefit (INR 3000) until the end of treatment. The median benefit amount was INR 0–500 for patients in the poorest quintile. The benefit helped ∼2% of study participants to remain below the catastrophic cost threshold. We estimated that a benefit amount of INR 10 000 for the whole duration of treatment (i.e. 6 months) would be able to push ∼43% of TB patients below the 20% catastrophic cost threshold.
- Our findings suggest that the level of social support is inadequate to improve the economic welfare of the patients and protect households with TB against catastrophic costs. We estimate that the appropriate level of benefit is as high as three times the current level of social protection provided. This, however, would put a significant strain on the already limited financing for TB. TB programmes therefore need to carefully consider targeted approaches to social protection. In addition, as the majority of treatment cost is incurred before starting the treatment, it is important that the social support/benefit is provided immediately after treatment registration.

## Introduction

Tuberculosis (TB) is one the leading causes of ill health and death worldwide (World Health Organization, 2022). The disease disproportionately affects the poor and marginalized groups mostly in their productive ages (Oxlade and Murray, 2012; World Health Organization, 2018). Households having TB patients find themselves in a spiral of poverty as high cost, huge income loss and several other economic consequences (e.g. borrowing/selling of personal belongings, inability to pay school fees, rents, regular bills etc.) are associated with TB treatment (Chatterjee *et al*., 2023; 2024). Further, TB and malnutrition have bidirectional relationships (Bhargava, 2016; Padmapriyadarsini *et al*., 2016). Therefore, developing strong socioeconomic support for TB-affected families is essential to reduce the burden of the disease; however, there are few examples of such support globally. Studies conducted in Brazil, Moldova, Peru and India concluded that TB-specific socioeconomic support can improve treatment success rates, however, in South Africa, providing vouchers to TB patients did not show much improvement in treatment outcomes in a real-world setting because of low implementation fidelity (Lutge *et al*., 2013; Ciobanu *et al*., 2014; Wingfield *et al*., 2017; Carter *et al*., 2019; Dave and Rupani, 2022). A recent Indian study showed that nutritional intervention can substantially reduce TB incidence among household contacts (Bhargava *et al*., 2023).

In 2021, 30 high TB burden countries accounted for 87% of global TB cases, and eight among those accounted for two-thirds of the burden, with India top of the list (World Health Organization, 2022). A recent study reported that 30–61% of TB patients faced catastrophic cost (defined as total TB treatment cost ≥20% pre-TB annual household income/expenditure) in India (Chatterjee *et al*., 2024), while the ‘End TB’ milestone was to achieve zero catastrophic cost for TB-affected households by 2020 (World Health Organization, 2015). The government of India introduced a benefit scheme namely ‘Nikshay Poshan Yojana’ (NPY) on 1 April 2018, with the objective of providing support mainly for nutrition to all notified TB patients (Government of India, 2018). A financial incentive of Indian rupees (INR) 500 [∼6 United States Dollars (US$6) at 2022 prices] per month was proposed to be either transferred directly to the registered beneficiaries’ validated bank accounts (direct benefit transfer—DBT) or could be distributed in-kind (nutritional supplements/food items) for the whole duration of anti-TB treatment. The first incentive of the benefit was supposed to be received on notification, the second one at 2 months after the end of intensive-phase (IP) follow-up results and for drug-susceptible (DS) TB patients, the final instalment at 6 months at the end-of-treatment follow-up results (Government of India, 2018). Overall, 22% of the total TB programme budget was estimated to be spent on this support per year from 2017 until 2020 (Central Tuberculosis Division, Directorate General of Health Services, 2016). As this is the only social support provided by the government to DS-TB patients, the question arises whether the amount is sufficient, not from a behavioural perspective but in terms of social protection, as a recent evaluation of a socio-economic intervention for TB patients in Peru found that social peer support was valued more highly than cash transfers (Evans, 2023).

Previous studies have shown various levels of coverage of NPY in different parts of the country. Dave and Rupani (2022) interviewed 426 DS-TB patients during January–September 2019 and reported that 91% of study participants received the benefit; however, 46% received the first instalment late and 49% received the last instalment after treatment completion. Using data collected from ‘Nikshay’ (a nationwide online platform that captures all the data of the registered TB patients), Patel *et al*. (2019) reported that 42.2% of patients received at least one instalment during April–September 2018, and Verma *et al*. (2023) found total benefit coverage of 76.81% during August 2020–September 2022. Jeyashree *et al*. (2024) analysed the national TB programme data of 3.7 million patients during 2018–2022 and found that the receipt of at least one instalment of the benefit increased from 57 to 76% over the study period. However, the median time the patients had to wait to receive the first instalment was 91 days in 2022, which was reduced from 200 days in 2018. Most of these studies used official data to estimate the benefit coverage. In this paper, we provide the reach, timing and amount of NPY benefit receipt by interviewing a cohort of 1482 notified adult DS-TB patients sampled from three different groups: general population (529 patients), urban slum dwellers (526 patients) and tea garden residents (427 patients) from four states, Assam, Maharashtra, Tamil Nadu and West Bengal, 16 districts and 118 TB units (one TB unit covers an average 200 000 population). As this study was embedded in a wider study on the economic impact of TB, we also examined the extent to which NPY benefit improved the economic welfare of TB patients (measured through reduction in catastrophic cost—a level of cost that is likely to result in a permanent negative economic impact on households) and estimated the potential amount of social support required to achieve a zero catastrophic-cost target.

## Materials and methods

### Study participants

Study participants from urban slum dwellers and tea garden areas were chosen as these two groups were defined as ‘high-risk groups’ in the national strategic plan 2017–2025 for TB in India, as poor living conditions, malnutrition and poverty put the people living in urban slums (a highly populated residential area with improper housing, sanitation and drinking water) and tea garden areas at high risk of getting the infection (Central Tuberculosis Division, Directorate General of Health Services, 2016). Participants were chosen from different groups to understand variation (if any) in treatment-seeking behaviour, cost of treatment and social protection requirements and to examine if any specific interventions are required for the high-risk groups during the process of TB elimination in the country. Methods of state selection and sample size calculations are reported in the supplementary Appendix and supplementary Table S1 (see online supplementary material).

### Interview schedule

Study participants who gave written informed consent were interviewed face-to-face at the intensive phase (IP), i.e. within 2 months of treatment, at the end of treatment and ∼7–8 months post-treatment, by 12 trained research assistants under the supervision of two core study team members during March 2019 to April 2023. During the COVID-19 period (March 2020 until July 2020), scheduled follow-up interviews were conducted over the telephone because of inter-state travel restrictions. Each telephone interview was also attended by one core team member.

### Study instruments

Study questionnaires were adapted from the World Health Organization’s TB patient cost survey (World Health Organization, 2017). Apart from understanding money and time spent for all activities related to the disease episode, during each interview, we asked study participants whether they had received any social welfare payment because of the disease, including NPY, and the amount received for each support. We examined the amount of NPY benefit receipt in relationship to wealth quintiles to explore the extent to which these subsidies reached the most vulnerable groups. Further, during end-of-treatment interviews, we asked how study participants spent the NPY benefit they received.

### Analysis of impact on catastrophic cost

Catastrophic cost was defined as total TB treatment cost (direct and indirect) ≥20% pre-TB annual household income (World Health Organization, 2017). Direct cost included money spent out of pocket on consultations, bed charges, drugs, laboratory/radiology tests, nutritional food/supplements, travel expenses and any other expenses. Indirect cost was calculated using both the human capital approach (HCA) and the output approach (OA) (World Health Organization, 2017). In the HCA, time spent by patient and guardian were calculated using the minimum wage rates of the respective states (https://paycheck.in/salary/minimumwages, 2024), while in the OA, difference in self-reported income in pre-treatment and during the treatment period was considered as indirect cost. The HCA measures lost productivity but does not capture indirect cost due to reduced productivity, as it multiplies the time spent while seeking and receiving care with wage rate (Chatterjee *et al*., 2024). On the other hand, as the OA captures income loss at different time periods, it captures reduced productivity but misses the time loss related to treatment. As these two approaches provide two different estimates, we used both methods while calculating the indirect cost.

During interviews in the IP, we asked all study participants about their monthly income (if applicable) and monthly household income for the month prior to diagnosis of TB. That was considered as pre-TB patient and household monthly income. We also asked them to report incomes at the time of each interview (IP and end-of-treatment). Those reported incomes were considered for estimating differences between pre-TB and during TB household incomes used in catastrophic cost calculation. For seasonal workers with wage fluctuations, we tried to ascertain average monthly income (World Health Organization, 2017). We examined catastrophic costs with and without the NPY benefit amount to estimate the extent to which the subsidies reduced catastrophic costs.

To estimate the potential level of NPY benefit amount required to reach zero catastrophic cost, we calculated the proportion of total TB treatment cost (direct and indirect) for the whole duration of TB treatment relative to pre-TB annual household income for each participant. Though the duration of DS-TB treatment is ∼6 months, many patients had an extended treatment period (especially patients with extrapulmonary TB, i.e. TB involving organs other than lungs). We considered costs incurred for the whole duration of the treatment while calculating total treatment cost. On the other hand, there were patients who died within the treatment period and discontinued their treatment. In those cases, total treatment cost was calculated until the date of death or date of last medicine taken. We then estimated the difference in treatment cost after adjusting for the 20% threshold of catastrophic cost. We considered only positive differences, as negative differences meant zero catastrophic cost. The adjusted positive difference of each participant was the amount of support that would be required to push the person below the 20% threshold of catastrophic cost. This exercise was conducted using treatment costs calculated using both indirect cost calculation methods: HCA and OA. We present the amount of support required using the HCA method of indirect cost calculation as the base case, and the results using the OA method are reported as sensitivity analysis. We also explored the sensitivity of the estimation of potential social support requirement to our assumptions about the proportion of cost using 15 and 25% catastrophic cost thresholds to provide ranges. While a 20% threshold is commonly used in TB, it is based on the level of cost associated with adverse TB outcomes (e.g. death, relapse, discontinuation of medicine) from Peru (Wingfield *et al*., 2014), which may not represent the Indian context. All costs and potential social support requirements are presented in 2022 INR and US$; 1 US$ = INR 78.5344.

## Results

### Characteristics of study participants

Most study participants were male (59–66%) and 42–57% were in the age group 18–34 years. Of study participants, 40% among tea garden residents, 15% of urban slum dwellers and 17% of the general population had no education. Overall, 61–67% of study participants were employed and 42–45% were the main source of household income. Average pre-TB monthly household income ranged from INR 9560 (US$122) [standard deviation (SD) 6707 (US$85)] for tea garden residents to INR 20 381 (US$260) [SD 18 526 (US$236)] for patients from the general population. Most study participants had bacteriologically confirmed pulmonary TB (56–61%); however, 25–31% had extrapulmonary TB, with plural effusion and cervical lymph node as the commonest forms. Of the study participants, 72 and 75% from the general population and urban slum dwellers, respectively, first visited private providers after symptom onset, while the same was true of only 31% of tea garden residents.

### NPY benefit

NPY benefits received at different phases and the median amounts received in each phase are given in Table 1. The number of participants decreased during end-of-treatment and post-treatment interviews because of missing patients for interviews. In supplementary Table S2, see online supplementary material, we present the number of participants who we were unable to interview at different phases and their reasons.

**Table 1.** NPY and other benefits for study participants at different phases of treatment

	General population	Urban slum dwellers	Tea garden residents
**Intensive-phase interviews**			
Total number of participants	529	526	427
Number of participants who received NPY benefit (%)	41 (7.7%)	31 (5.9%)	3 (0.7%)
Median amount of NPY benefit received (INR)	1000	1000	500
Number of participants who got paid sick leave (%)	8 (1.5%)	7 (1.3%)	0 (0.0%)
Median amount received in 1 month (INR)	19 848	3330	0
Number of participants who received food support (%)	18 (3.4%)	37 (7.0%)	25 (5.8%)
Median amount received in 1 month (INR)	2000	2500	150
Number of participants who received other support (%)	19 (3.6%)	12 (2.3%)	12 (2.8%)
Median amount received in 1 month (INR)	3000	3000	1000
**End-of-treatment interviews**			
Total number of participants^a^	497	499	396
Number of participants who received NPY benefit (%)	225 (45.3%)	239 (47.9%)	118 (29.8%)
Median amount of NPY benefit received (INR)	2000	1500	1500
Number of participants who did not check the receipt of NPY benefit	64 (12.9%)	53 (10.6%)	69 (17.4%)
Number of participants who did not know about the receipt of NPY benefit	18 (3.6%)	26 (5.2%)	13 (3.3%)
Number of participants who did not receive NPY benefit for other reasons^b^	5 (1.1%)	0 (0%)	13 (3.3%)
Number of participants who got paid sick leave (%)	0 (0.0%)	0 (0.0%)	0 (0.0%)
Number of participants received INR 3000 as NPY benefit	55 (11.1%)	43 (8.6%)	21 (5.3%)
Number of participants who received food support (%)	18 (3.6%)	58 (11.6%)	48 (12.1%)
Median amount received in 1 month (INR)	650	572	200
Number of participants who received other support (%)	10 (2.0%)	8 (1.6%)	9 (2.3%)
Median amount received in 1 month (INR)	3250	6750	4000
**Post-treatment interviews**			
Total number of participants^a^	464	447	379
Number of participants who received NPY benefit (%)	49 (10.6%)	24 (5.4%)	101 (26.6%)
Median amount of NPY benefit received (INR)	2000	2000	2500
Number of participants who did not check the receipt of NPY benefit	24 (5.1%)	20 (4.5%)	40 (10.6%)
Number of participants who did not know about the receipt of NPY benefit	9 (1.9%)	20 (4.5%)	16 (4.2%)

Note: 1 US$ = INR 78.5344. ^a^Number of participants decreased because of loss to follow-up.

b Other reasons include did not submit documents (general population)/did not have bank account/bank account not active (tea garden residents).

IP interviews were conducted after a median 29 days of starting TB treatment, end-of-treatment interviews were conducted after a median 127 days from IP interviews, and post-treatment interviews were conducted after a median 222 days from treatment completion. During end-of-treatment interviews, 53 and 54% of study participants from the general population and urban slum dwellers, respectively, reported having received NPY benefit, with the median amount received being INR 2000 (US$25) for participants in the general population and INR 1500 (US$19) for urban slum dwellers (Table 1). The proportion of participants who reported receiving benefit at the end of treatment in tea garden areas was much lower, ∼31%. The full amount of benefit until treatment completion [INR 3000 (US$38)] was received by ∼5% of participants in tea garden areas, 9% of urban slum dwellers and 11% of the general population. About 27% of participants from tea garden areas reported having received the benefit after completing treatment (Table 1).

Apart from NPY benefit, study participants received few other benefits, such as paid sick leave and food support (given mostly in kind, which we monetized) from neighbours, friends, relatives, employers and non-government organizations. Table 1 shows that few study participants received other benefits.

**Figure 1.**
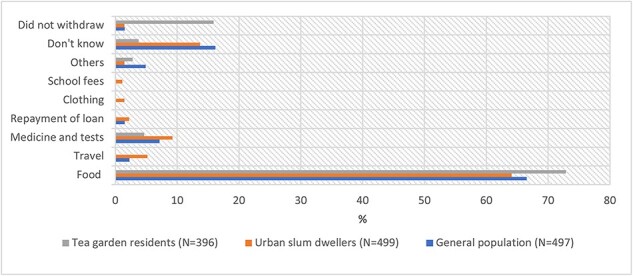
Usage of NPY benefit by study participants reported at end-of-treatment interviews

Our results on how the NPY benefit was spent are reported in (Figure 1). The majority used the benefit for purchasing food items, while some used it for medicines and tests, travel expenses and other purposes. Of the participants, 14% among urban slum dwellers and 16% among the general population were not aware of the receipt of the benefit, while 16% of participants among tea garden residents did not withdraw the money (Figure 1). The reasons for not withdrawing were difficulty in travelling to the banks, as that would cause a day’s income loss, and huge travel expenses for the participants as they live in remote rural areas.

In Table 2, we present the amount of NPY benefit received by demographic and socioeconomic characteristics, and the proportion of participants who faced catastrophic cost with and without NPY benefit, using the HCA method of indirect cost calculation. For all groups, the median amount of NPY benefit received was less for those in older age groups, who had lower education and who belonged to the poorest quintile (Table 2). Using the total amount of NPY benefit received by each participant until end-of-treatment interviews, we examined how NPY benefit helped study participants in mitigating economic burden. We found that the benefit helped 25 (∼2%) of study participants to remain below the catastrophic cost threshold (Table 2).

**Table 2.** NPY benefit and catastrophic cost for study participants by socio-economic characteristics

	General population				Urban slum dwellers				Tea garden residents			
	Number of participants	Median (IQR) amount of NPY received until end of treatment (INR)	Catastrophic cost without NPY, N (%)	Catastrophic cost with NPY, N (%)	Number of participants	Median (IQR) amount of NPY received until end of treatment (INR)	Catastrophic cost without NPY, N (%)	Catastrophic cost with NPY, N (%)	Number of participants	Median (IQR) amount of NPY received until end of treatment (INR)	Catastrophic cost without NPY, N (%)	Catastrophic cost with NPY, N (%)
**Age**												
18–24 years	97	500 (0, 1500)	27 (27.8)	25 (25.8)	115	500 (0, 2000)	35 (30.4)	34 (29.6)	114	1000 (0, 2000)	52 (45.6)	49 (43.0)
25–34 years	127	1000 (0, 2000)	29 (22.8)	27 (21.3)	117	500 (0, 1500)	35 (29.9)	35 (29.9)	130	0 (0, 1875)	47 (36.2)	45 (34.6)
35–44 years	116	1000 (0, 2500)	36 (31.0)	34 (29.3)	105	1000 (0, 2000)	35 (33.3)	34 (32.4)	90	1000 (0, 2875)	44 (48.9)	38 (42.2)
45–54 years	89	1000 (0, 2000)	25 (28.1)	25 (28.1)	95	1000 (0, 2000)	21 (22.1)	20 (21.1)	47	0 (0, 1000)	17 (36.2)	16 (34.0)
55–64 years	66	750 (0, 2000)	27 (40.9)	25 (37.9)	73	500 (0, 2000)	29 (39.7)	27 (37.0)	36	500 (0, 1500)	15 (41.7)	15 (41.7)
≥65 years	34	750 (0, 2000)	12 (35.3)	12 (35.3)	21	0 (0, 2000)	6 (28.6)	6 (28.6)	10	0 (0, 0)	7 (70.0)	7 (70.0)
**Sex**												
Male	348	1000 (0, 2000)	98 (28.2)	91 (26.1)	308	500 (0, 2000)	73 (23.7)	71 (23.1)	253	0 (0, 2000)	94 (37.2)	88 (34.8)
Female	181	500 (0, 2000)	58 (32.0)	57 (31.5)	218	500 (0, 2000)	88 (40.4)	85 (39.0)	174	500 (0, 2000)	88 (50.6)	82 (47.1)
**Education**												
Did not attended school	88	500 (0, 2000)	38 (43.2)	38 (43.2)	81	0 (0, 1500)	33 (40.7)	31 (38.3)	170	500 (0, 2000)	74 (43.5)	70 (41.2)
Primary education	110	500 (0, 2000)	31 (28.2)	30 (27.3)	84	500 (0, 1500)	27 (32.1)	26 (31.0)	145	1000 (0, 2000)	63 (43.4)	55 (37.9)
Secondary education	167	1000 (0, 2000)	51 (30.5)	46 (27.5)	243	1000 (0, 2000)	64 (26.3)	62 (25.5)	81	0 (0, 1000)	36 (44.4)	36 (44.4)
Higher secondary education	78	1000 (0, 2000)	16 (20.5)	15 (19.2)	66	500 (0, 2000)	21 (31.8)	21 (31.8)	25	1000 (0, 2000)	8 (32.0)	8 (32.0)
Graduate and above	86	1500 (0, 2000)	20 (23.3)	19 (22.1)	52	1500 (0, 2125)	16 (30.8)	16 (30.8)	6	1000 (250, 1000)	1 (16.7)	1 (16.7)
**Wealth quintile**												
Poorest	77	500 (0, 2000)	25 (32.5)	24 (31.2)	63	0 (0, 1500)	25 (39.7)	24 (38.1)	157	0 (0, 2000)	74 (47.1)	69 (43.9)
Poorer	98	1000 (0, 2000)	41 (41.8)	37 (37.8)	117	0 (0, 1500)	34 (29.1)	33 (28.2)	81	500 (0, 1500)	35 (43.2)	34 (42.0)
Middle	81	500 (0, 2000)	30 (37.0)	29 (35.8)	107	500 (0, 1750)	40 (37.4)	39 (36.4)	108	0 (0, 2000)	36 (33.3)	32 (29.6)
Richer	105	1000 (0, 2000)	31 (29.5)	30 (28.6)	122	1000 (0, 2000)	31 (25.4)	29 (23.8)	69	500 (0, 2000)	33 (47.8)	31 (44.9)
Richest	168	1000 (0, 2000)	29 (17.3)	28 (16.7)	117	1000 (0, 2500)	31 (26.5)	31 (26.5)	12	0 (0, 250)	4 (33.3)	4 (33.3)

Note: Catastrophic cost was calculated using the HCA method of indirect cost calculation. IQR = Inter-quartile range. 1 US$ = INR = 78.5344.

### Cost of TB treatment

Using the HCA as an indirect cost calculation method, total TB treatment cost was INR 29 960 (US$381) [SD 58 781 (US$748)] for participants in tea garden areas, INR 30 782 (US$392) [SD 44 061 (US$561] for urban slum dwellers and INR 32 829 (US$418) [SD 49 889 (US$635)] for participants in the general population. About 60% of the cost was incurred in the pre-diagnosis phase for participants among the general population and urban slum dwellers, while for tea garden residents the proportion of pre-diagnosis cost to total cost was 44%.

### Estimation of potential social support requirement

**Figure 2.**
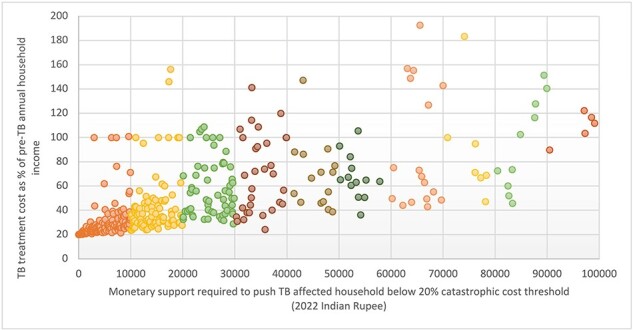
Requirement of social support to push tuberculosis patients below the 20% threshold of catastrophic cost (using the HCA of indirect cost calculation)

We combined all groups of participants to estimate the potential amount required as social support to push TB patients below the 20% threshold of catastrophic cost. Using the HCA method of indirect cost calculation, ∼34% (499/1482) faced catastrophic cost without adjusting for NPY benefit amount. We excluded 58 (4%) study participants while calculating the benefit amount, as for 52 study participants the difference of treatment cost and the 20% threshold was >INR 100 000 (US$1273), and for 6 study participants the proportion of treatment cost to pre-TB annual household income was >200%. Our estimate showed that a benefit amount of INR 10 000 (US$127) for the whole duration of treatment (i.e. 6 months) could push ∼43% of TB patients who faced catastrophic cost back below the 20% threshold (Figure 2). If the benefit amount increased to INR 20 000 (US$255) for 6 months, it could push back ∼61% of TB patients, while an amount of INR 30 000 (US$382) for 6 months of treatment could push back ∼74% of patients.

Using the UCA, 498 study participants faced catastrophic cost. However, the graph was plotted considering 440 study participants as for 52 study participants, the difference in treatment cost after adjusting for the 20% threshold was > INR 100 000; and for 6 participants, the proportion of treatment cost to pre-TB annual household income was > 200%; 1 US$ = INR 78.5344

### Sensitivity analysis

**Figure 3.**
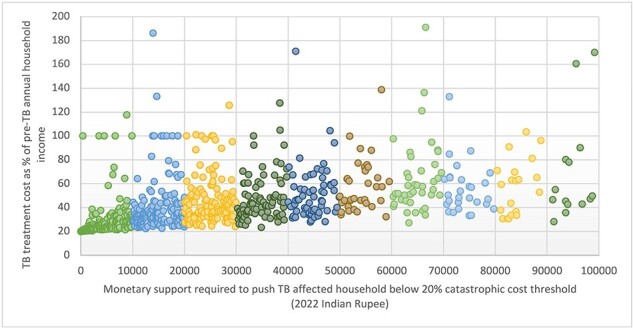
Requirement of social support to push tuberculosis patients below the 20% threshold of catastrophic cost (using the OA of indirect cost calculation)

Using the OA method of indirect cost calculation, 893/1482 (60%) of study participants faced catastrophic cost without NPY benefit adjustment. Using this method, a benefit amount of INR 10 000 (US$127) for 6 months of treatment could push a much lower proportion of TB patients (27%) below the 20% threshold as compared to the HCA method (43%), as the proportion of patients facing catastrophic cost was much higher using the OA (60%) compared to the HCA (34%) (Figure 3). A benefit amount of INR 20 000 (US$255) could push back ∼49% of TB patients while ∼64% of patients would be below the catastrophic cost threshold with a benefit amount of INR 30 000 (US$382) for 6 months.

Using the HCA, 893 study participants faced catastrophic cost. However, the graph was plotted considering 815 study participants as for 75 study participants, the difference in treatment cost after adjusting for 20% threshold was . INR 100 000; and for 3 participants, the proportion of treatment cost to pre-TB annual household income was > 200%; 1 US$ = INR 78.5344

Using the HCA method of indirect cost calculation and a 15% catastrophic cost threshold, a potential benefit amount of INR 10 000 (US$127) for 6 months of treatment could push ∼43% of patients who faced catastrophic cost below the catastrophic cost threshold, while at a 25% threshold, the same amount of benefit could push ∼40% patients below the threshold. Using the OA method of indirect cost calculation, ∼INR 1700 (US$22) support per month for 6 months could push 25 (at a 15% threshold) to 15% (at a 25% threshold) of patients below the catastrophic cost threshold.

## Discussion

The government of India introduced the NPY scheme in 2018 with the objective of providing support mainly for nutrition to all notified TB patients. This is the only TB-specific social benefit scheme available for DS-TB patients in India. Our study, following a cohort of 1482 adult DS-TB patients divided into three groups: general population, urban slum dwellers and tea garden residents from 118 TB units of 16 districts of four Indian states, showed that the uptake of this benefit was modest; however, the majority of participants who received the benefit spent the amount for food. On the other hand, ∼16% of study participants in tea garden areas did not withdraw the benefit, as reported during end-of-treatment interviews, because travelling to banks implied a day’s wage loss and huge travel expenses. The difficulties in accessing benefits were also reported in another study which highlighted patients’ inability to visit banks during illness (Patel *et al*., 2019). Overall, 16% of participants among the general population and 14% among urban slum dwellers reported that they did not know whether they had received the benefit. This could be either because they did not put much importance on the benefit or because other family members handled the benefit (in the case of students, old patients, homemakers) and hence, the study participants were not aware of it.

We found that 53% of study participants among the general population, 54% among urban slum dwellers and 31% among tea garden residents received NPY benefit during end-of-treatment interviews which were conducted after ∼156 days from the initiation of TB treatment. The median amount of benefit received was INR 1500 (US$19) for participants among urban slum dwellers and tea garden residents and INR 2000 (US$25) for participants among the general population. As per the government notification, the first instalment of the benefit was supposed to be received immediately after registration for treatment (Government of India, 2018); however, only 3/427 (0.7%) participants among tea garden residents reported receiving the benefit after 1 month of treatment initiation while 6–8% of participants in other groups received the benefit at that time. Therefore, the majority of study participants did not receive the benefit when they needed it most. This finding is in line with a recently published study that evaluated the NPY scheme using national TB programme data of 3.7 million patients over 5 years (Jeyashree *et al*., 2024). They reported that the median time to receive the first instalment was reduced from 200 days in 2018 to 91 days in 2022.

The proportions of TB patients reported to receive NPY benefits in our study were lower compared to other studies. This is probably because we relied on patient reporting, which may be an underestimate, while other studies used programme data (Verma *et al*., 2023; Jeyashree *et al*., 2024). Our results were also lower compared to another study which relied on patient reporting (Dave and Rupani, 2022). This could be because the other study covered patients from only 1 district while our patients were from 16 districts across four states. It was evident from our study that many patients living in remote rural areas did not receive the benefit, therefore overall uptake of the benefit in our study was lower. Although Dave and Rupani (2022) reported that 91% of study participants received the benefit, however only ∼37% received it during the treatment period while 54% received it after treatment completion. This finding is similar to our findings which showed that many TB patients received NPY benefit after treatment completion.

Our study found that 34–60% of study participants faced catastrophic cost depending on indirect cost calculation methods, and the NPY amount was able to push only ∼2% of study participants below the 20% catastrophic cost threshold using the HCA method of indirect cost calculation. Therefore, a substantial amount of benefit is required to ensure zero catastrophic cost for TB-affected households in India. Our estimate showed that a support amount of INR 10 000 (US$127) for a 6 month treatment period would help 43% of study participants who faced catastrophic cost to achieve zero catastrophic cost when treatment cost was calculated using the HCA method of indirect cost calculation, while this proportion is 27% using the OA method of indirect cost calculation. The proportion was much lower using the OA, as income loss associated with a TB episode was very high among our study participants and hence, the proportion facing catastrophic cost was also very high (60%) as compared to the HCA method (34%). Though increasing the benefit amount could be an immediate short-term solution to protect TB patients from catastrophic cost, the government should focus on reducing the cost and the massive income loss associated with TB treatment as a long-term measure. The government took several initiatives such as active case finding, usage of high-efficiency diagnosis tools and private sector engagement for early case detection (Central Tuberculosis Division, Directorate General of Health Services, 2016); however, our study showed that ∼60% of total TB treatment cost was borne before the diagnosis phase, which clearly emphasizes that these initiatives need to be further strengthened along with focusing on demand-side initiatives (e.g. active community engagement), which have not received adequate attention to date.

Increasing the benefit amount from INR 3000 (US$38) to INR 10 000 (US$127) for 6 months of treatment would have a significant impact on the TB programme budget. As 34–60% of TB patients faced catastrophic cost, to manage this the government may think of a targeted approach for social support, but should note the challenges of income/means-tested targeting, including potential inequities as those most in need can struggle to complete the means testing required. In this situation, targeting of specific populations may be an option. For example, TB patients from tea garden areas could be the targeted group as our study showed they had very low income, a significant proportion of them were in poorest quintiles, more patients faced catastrophic cost and the death rate among them was higher as compared to the other two groups. Further, their poor nutritional status has been reported in the literature (Yasmin *et al*., 2022). This group also received the minimum amount of NPY benefit among all the study groups. Therefore, supporting them with the increased benefit amount could be the first step towards a targeted approach.

A recent review on experiences of conditional and unconditional cash transfers in improving health service use and outcomes for different health conditions reported that the recipients perceived cash transfers to be helpful for immediate necessities and enhanced the empowerment, autonomy and agency, especially of women. However, they also felt that the amount given was too little in relation to their total needs (Yoshino *et al*., 2023). Our study results corroborate these findings as we also found that the NPY benefit was helpful as study participants mostly used it for buying food; however, the amount was not enough to push TB-affected households below the 20% catastrophic cost threshold. A modelling study reported that cash-transfer only would not be enough to achieve the zero catastrophic cost target in countries like India, as indirect cost associated with TB is high (Verguet *et al*., 2017). Our study supports this finding—the proportion of patients facing catastrophic cost using the OA was much higher as compared to the same using the HCA. This clearly implies that there is a huge income loss associated with TB, and therefore other social protection in terms of paid sick leave, ensuring uninterrupted livelihood during TB disease and better insurance coverage also need consideration.

Although our study provides a detailed understanding on the status of NPY benefit for different groups of TB patients and the potential amount of social support required to achieve zero catastrophic cost for TB patients in India, it has the following limitations. First, NPY status was recorded as per patient reporting and was not cross-checked with any official documents. Therefore, there could be recall bias; however, as many patients were unable to identify the record of benefit receipt in their bank passbooks and requested the research team for help during interviews, we helped them identifying the same. In that way, a proportion of records was cross-checked. However, as that was not part of our proposed work, we did not record this activity. Hence, we are unable to report the proportion of bank passbooks cross-checked for validation. Second, the estimated amount of support required to achieve zero catastrophic cost was estimated using data collected from notified DS-TB patients who received treatment from public facilities. This amount will probably increase if muti-drug TB patients and patients treated in the private sector are added in the calculations. Finally, pre-TB annual household income was the denominator in the catastrophic cost calculation. It is well recognized that there could be bias in reporting of income and/or it could be under-reported. However, as the majority of our study participants and the household members were daily wage earners, we did not notice any hesitancy while reporting income. Further, as we collected income data during all rounds of interviews, we cross-checked and found that the reporting was consistent.

## Conclusions

Our study has several policy implications. First, it was evident that the support provided through NPY was not sufficient to help TB patients in managing their financial distress. Although NPY was meant mainly for nutritional support, given the high proportion of households facing catastrophic cost, government needs to consider revising the amount for social support and/or other ways to protect patients from the devastating effects of TB. The insufficiency of benefit amount was also reported by patients in another recent study (Verma *et al*., 2023) Second, as per the present guideline, NPY benefit should be given in three instalments. However, as the majority of treatment cost was incurred before starting the treatment, we recommend that at least half of the revised benefit amount should be given immediately after registration of the patient so that they can mitigate part of the expenses incurred before treatment initiation. Third, as a significant proportion of the study participants did not receive any benefit until the end-of-treatment interviews, and other studies have noted several administrative challenges in the process (Dave and Rupani, 2022; Verma *et al*., 2023), solving these challenges should also be a priority. Fourth, as the majority of patients who received the benefit used it for food and also, as the poorest among the groups, the tea garden residents, who needed the benefit the most, could not visit banks several times to check/withdraw the money, government may consider providing TB patients with vouchers at the time of registration and during TB drug pick-ups and liaison with the local public distribution system, so that the patients can collect additional rations from the stores. Further, as efforts are ongoing for adding millets in the public distribution system (Mohanty and Mohanty, 2023), distributing those to TB patients will be particularly helpful. Government has recently introduced the ‘Nikshay Mitra’ scheme, where elected representatives, non-government organizations and individuals can support TB patients throughout their treatment period (https://dashboards.nikshay.in/community_support/overview_dashboard). As our study showed people living in remote rural areas and tea garden areas need more support, this scheme may also prioritize helping those groups. Finally, revision of the amount of social support and timing of benefit distribution are immediate solutions to protect TB patients from financial distress; however, the long-term goal should be to identify ways to reduce pre-diagnosis costs so that overall treatment cost and hence, catastrophic cost, can be reduced.

## Supplementary Material

czae065_Supp

## Data Availability

The data underlying this article will be shared on reasonable request to the corresponding author.

## Reflexivity statement

The lead and corresponding author, a program lead: health economics (woman), along with the research fellow (man) are from India, where the study was conducted, and the other two authors are from high income countries: America (man) and The Netherlands (woman). The second author, a global lead of a TB private-sector engagement programme, and the senior author, a professor in health economics, have extensive experience in working in TB in many low- and middle-income countries including India, while the first and third authors have vast work experience in India.
