# Supplementary material for: Estimation of potential social support requirement for tuberculosis patients in India
Source: Health Policy Plan. 2024 Jul 10;39(8):854–63. doi: 10.1093/heapol/czae065 (PMC11384111; doi:10.1093/heapol/czae065)
Supplement: czae065_Supp [file czae065_supp.zip › Supplementary appendix_Revised.docx]

**Estimation of potential social support requirement for tuberculosis patients in India**

***Supplementary Appendix***

*Sampling strategy*

For the costing study, national representativeness can be achieved by considering a limited number of states selected using an appropriately stratified sampling design. The World Health Organization Study on global AGEing and adult health (SAGE) sampling design was followed in this context (<https://www.who.int/data/data-collection-tools/study-on-global-ageing-and-adult-health>). As a first step, 29 states of India were stratified into six levels of development and six geographic locations. A composite development index was constructed using the following indicators at the state level: Infant mortality rate; female literacy rate; full immunization coverage rate; and per capita income. Principal component analysis technique was used to construct the composite index. Quantiles were then used to categorize the states into six levels of development (*Table S1*). Based on the availability of study budget, Assam, Maharashtra, Tamil Nadu, and West Bengal were selected for this study as these states not only represent different levels of development but also cover different regions of the country. Apart from that, Assam, Tamil Nadu, and West Bengal have substantial land area for tea gardens, and Maharashtra, Tamil Nadu and West Bengal are among the top five states in terms of urban slum population in the country. TB patients from general population were drawn from all four states.

In the next stage of sampling, 3-5 districts from each state were selected purposively based on the dominance of the high-risk groups. From the sampled study districts, TB units (TUs - one TU covers 200,000 population (range 150,000 - 250,000) for rural and urban areas, 100,000 (range 75,000 - 150,000) in hilly, tribal, and difficult areas) were then identified which cater to the study high-risk groups. As all TUs cater to general population, there was no specific identification of TUs for covering patients from general population.

*Patient recruitment*

In the next step, from all identified TUs, adult (18 years and above) TB patients who were at their intensive phase of treatment (DS-TB requires a minimum of six months of treatment, of which the first two months are called the intensive phase and the following four months the continuation phase) during the visit of the study team and gave written informed consent to participate in the study were interviewed.

*Sample Size for each group for the main study*

Number of DS-TB patients required in each group i.e., from general population, tea garden areas and urban slum dwellers was calculated to estimate the mean cost, if the resulting estimate is to fall within 7% of the true estimate with 95% confidence. The following formula was used to derive the number of DS-TB patients that must be sampled in each group

$$n=Z_{1-\alpha/2}^{2}\frac{\sigma^{2}}{(ɛ.{\mu)}^{2}}DEFF$$

where $\sigma$ is the population standard deviation, $\mu$ is the population mean, $\epsilon$ is the relative precision,  $Z_{1-\frac{\alpha}{2}}$ is the $\left( 1-\frac{\alpha}{2} \right)^{th}$ quantile of the standard normal distribution and DEEF is the design effect to account for the cluster sampling design. Design effect helps account for the clustering in costs borne by patients from the same TU. An earlier study conducted in Tamil Nadu state in India (John *et al*. 2009), reported a mean cost of Indian Rupee (INR) 23,991 along with a standard deviation of INR 12,258 (converted in 2015 prices). Therefore, $\mu$= INR 23,991 and $\sigma$= INR 12,258 were assumed. For the resulting estimate to fall within 7% of the true estimate with 95% confidence, $\epsilon$=7% and $\alpha$=0.05, i.e.  $Z_{1-\frac{\alpha}{2}}=1.96$ were set. For most demographic health surveys, the design effect, which is the ratio of variances under cluster sampling and simple random sampling, comes out to be less than or equal to 2. Therefore, DEFF was set as 2. Based on these specifications, 410 DS-TB patients are required to estimate the mean cost within 7% of the true estimate with 95% confidence accounting for survey design. Considering a 10% loss to follow-up and another 10% non-response, the final sample size of TB patients required was 512 in each group. This implies that 1,536 DS-TB patients (512 patients in each group x three groups: general population, tea garden areas and slum dwellers) were required to be interviewed in total for the main study. 512 patients in each group were sampled from all four states except for patients in tea garden areas who were sampled from three states as the state Maharashtra does not have tea gardens.

**References**

John KR, Daley P, Kincler N, Oxlade O, Menzies D. Costs incurred by patients with pulmonary tuberculosis in rural India. Int J Tuber Lung Dis. 2009; 13: 1281-1287.

World Health Organization. WHO's Study on global AGEing and adult health (SAGE).

[https://www.who.int/data/data-collection-tools/study-on-global-ageing-and-adult-health. Accessed January 2](https://www.who.int/data/data-collection-tools/study-on-global-ageing-and-adult-health.%20Accessed%20January%202), 2024.

**Table S1: States stratified based on levels of development and regions**

|  | Levels of development | | | | | |
| --- | --- | --- | --- | --- | --- | --- |
| Regions | I (Most developed) | II | III | IV | V | VI (least developed) |
| North | Delhi | Himachal Pradesh, Punjab | Haryana, Uttarakhand |  | Jammu & Kashmir |  |
| Central |  |  |  |  | Chhattisgarh | Madhya Pradesh, Uttar Pradesh,  Rajasthan |
| East |  |  | **West Bengal** |  | Odisha,  Jharkhand | Bihar |
| North-east | Sikkim | Mizoram  Tripura | Manipur | Nagaland  Arunachal Pradesh | Meghalaya | **Assam** |
| West | **Maharashtra**  Goa |  |  | Gujarat |  |  |
| South | Kerala | **Tamil Nadu** | Karnataka | Andhra Pradesh |  |  |

**Table S2: Reasons for missed interviews in different treatment phases.**

| **Study participants** | **Intensive phase - N** | **End of treatment follow-up – N** | **Reasons – N (%)** | **Post- treatment follow-up – N** | **Reasons – N (%)** |
| --- | --- | --- | --- | --- | --- |
| General population | 529 | 497 | Death – 13 (2.46%)  Refusal – 9 (1.70%)  No trace – 6 (1.13%)  Migrate out – 1 (0.19%)  Others* – 3 (0.57%)  Total – 32 (6.05%) | 464 | Death – 10 (2.01%)  Refusal – 12 (2.41%)  No trace – 11 (2.21%)  Total – 33 (6.63%) |
| Urban slum dwellers | 526 | 498 | Death – 7 (1.33%)  No trace – 13 (2.47%)  Refusal – 7 (1.33%)  Regimen change – 1 (0.19%)  Total – 28 (5.32%) | 444 | Death – 16 (3.21%)  No trace – 23 (4.21%)  Refusal – 15 (3.01%)  Total – 54 (10.42%) |
| Tea garden residents | 427 | 396 | Death – 22 (5.15%)  No trace – 5 (1.17%)  Regimen change – 2 (0.47%)  Migrate out – 2 (0.47%)  Total – 31 (7.26%) | 379 | Death – 10 (2.52%)  No trace – 6 (1.52%)  Refusal – 1 (0.25%)  Total – 17 (4.29%) |

Notes: *Others include misdiagnosis, severe illness
